# First report of *Przhevalskiana silenus* derived recombinant hypodermin C based indirect ELISA for serodiagnosis of goat warble fly myiasis

**DOI:** 10.1038/s41598-022-17760-5

**Published:** 2022-08-04

**Authors:** Anish Yadav, Shafiya Imtiaz Rafiqi, Vikas Yadav, Anand Kushwaha, Rajesh Godara, Shilpa Sood, Mohd Altaf Bhat, Rajesh Katoch, Rosario Panadero-Fontán

**Affiliations:** 1 Division of Veterinary Parasitology, Faculty of Veterinary Sciences and Animal Husbandry, Sher-E-Kashmir University of Agricultural Sciences and Technology of Jammu, RS Pura, Jammu, Union Territory of Jammu and Kashmir 181102 India; 2 Division of Veterinary Pathology, Faculty of Veterinary Sciences and Animal Husbandry, Sher-E-Kashmir University of Agricultural Sciences and Technology of Jammu, RS Pura, Jammu, Union Territory of Jammu and Kashmir 181102 India; 3 Division of Veterinary Microbiology and Immunology, Faculty of Veterinary Sciences and Animal Husbandry, Sher-E-Kashmir University of Agricultural Sciences and Technology of Kashmir, Shuhama, Srinagar, Union Territory of Jammu and Kashmir 190006 India; 4 INVESAGA Group, Department of Animal Pathology, Universidade de Santiago de Compostela, 27002 Lugo, Spain

**Keywords:** Biochemistry, Biological techniques, Biotechnology, Immunology, Molecular biology, Zoology

## Abstract

Goat warble fly infestation (GWFI) is a subcutaneous myiasis caused by larvae of *Przhevalskiana silenus*, an insect belonging to the order Diptera*.* The diagnosis of GWFI is challenging in the early larval instars (L1 and L2) as they are occult under the skin and hair coat causing prolonged economic loss in form of meat and hide damage. This necessitates early diagnosis for disease control at herd level and its prophylactic management to prevent economic losses. Hypodermins, a class of serine proteases from Hypoderminae subfamily have been used as serodiagnostic antigens for the past four decades for diagnosis of warble fly myiasis. In this study,the immunodominant antigen Hypodermin C (HyC) from *P. silenus* has been recombinantly expressed in *E. coli* and immunogenic characterisation of expressed protein was done. The protein shows hallmark residues in conserved cysteine and catalytic triad typical of serine proteases along with similar profile of immunoreactivity towards Hypoderminae infestation. The present study reports an optimised indirect-ELISA based on recombinant HyC derived from *P. silenus* for early diagnosis of GWFI. The optimised indirect ELISA provides a sensitive and specific immunodiagnostic for mass surveillance of the GWFI with diagnostic specificity and sensitivity of 96% and 100%, respectively and not showing any cross reactivity against other important parasitic and bacterial diseases of goats. This study presents the first report of indirect ELISA based on recombinant Hypodermin C antigen derived from *P. silenus* for the serosurveillance of goat warble fly disease*.*

## Introduction

Goat warble fly infestation (GWFI) is a subcutaneous myiasis caused by larvae of *P. silenus*, an oestrid fly belonging to the order Diptera, family Oestridae, subfamily Hypoderminae^1^. The disease is characterised by the presence of subcutaneous warbles on the dorsum and lumbar region of domestic and wild ruminants. GWFI occurs in many countries with considerable difference throughout the world due to geographical and meteorological variation and grazing pattern^2–4^. The disease is most prevalent in Mediterranean and Indian subcontinent in subtropical climatic zones ranging from northern India, Pakistan, Iran, Turkey and Southern Italy^5–9^. In India, the disease is prevalent in the northwestern Himalayan region, especially, in Bakerwali breed goats. The larvae infest the skin at lumbar and flank regions of goats causing sustained economic loss in form of reduced weight gain, hide value depreciation and degradation of meat quality by carcass trimming loss upon slaughter^7,10,11^. The GWFI is peculiar in the infestation as all the stages of larvae infest subcutaneous region of dorsum similar to *H. acateon* unlike other members of subfamily Hypoderminae (*Hypoderma bovis* and *H. lineatum)* which migrate to internal locations in the host^12,13^.

The life cycle of goat warble fly permits infestation of larvae for about 7–9 months within the host. The larval stages of *P. silenus* range in size from 2 to 7.9 mm at L1 stage, 8–9.9 mm at L2 stage and 10–18 mm L3 stage which all inhabit the subcutaneous region of dorsum until falling off the host for pupation in ground soil^7,12^.

The host infestation by first instar larvae remains occult from physical detection or visual observation by the farmer due to small size of L1 and subcutaneous presence without the formation of palpable warbles, externally on the dorsum. The infestation of advanced larval stages causes irreversible loss to hide and meat through L2 and L3 instars^7,10,12^.

The diagnosis of GWFI is mainly based on physicoclinical observation by palpation of warbles on the dorsum which are evident only after second and third larval instars have developed which occurs about 5–9 months after infestation. This has promoted the development of serological assays for the early diagnosis of hypoderminae infestation. Hypoderminae insects are known to possess three main serine proteases viz. Hypodermin A (HyA), Hypodermin B (HyB) and Hypodermin C (HyC). Hypoderminae origin HyC is a member of collagenolytic enzymes related to the trypsin family. The HyC is primarily secreted by L1 larvae to degrade the collagen at physiological conditions while entering the host tissue^14–16^, whereas HyA and HyB serve as immunomodulators to suppress host immune response and promote larval survival in the host^17,18^. The molecule of HyC has been characterised as a major immunodominant antigen and suitable candidate for detecting *Hypoderma* specific antibodies from cattle and other ruminants^19–21^.

The available serological tests have been based on *Hypoderma* spp antigens, primarily, from *H. lineatum* and *H. bovis*^22–28^.

The native HyC from *H. lineatum* and *H. bovis* has been widely used as antigen for serodiagnosis of hypodermosis in cattle population in several countries^29–31^. The recombinant hypodermin C (rHyC) from *H. lineatum* and *H. bovis* have been produced in heterologous expression system and used as a diagnostic antigen for detection of antibody from cattle sera^26–28^. The detailed molecular analyses of HyC has been performed to gain the information on sequence and biochemical features of HyC derived from *H. lineatum*^15,32^.

The comparative utility of rHyC antigen has been assessed against using native antigen preparation from *H. lineatum* and has been shown as effective alternative to the native antigen or crude preparations derived from the larval lysates in diagnostic application^33^. In a separate study, the comparative utility of purified HyC antigen has been shown over the use of crude lysate extract for detecting anti-*Hypoderma* antibodies in cervids^34^.

The cross reactivity of HyC from *H. lineatum* and *H. bovis* origin has been established and it is utilized for the diagnosis of other related *H. sinense*, *H. actaeon*, *H. tarandi* and *P. silenus*^21,35–38^. However, in India due to religious issues collection of *Hypoderma* species larvae from cattle is not possible and thus serodiagnosis of Hypoderminae cannot be achieved using native antigen and have to depend on costly commercial diagnostic kits. So, exploration of an antigen obtained from other Hypoderminae species different than *H. lineatum* and *H. bovis* is required. Moreover, at global level till date *P. silenus* antigen has not been explored for serodiagnosis of goat warble fly and other hypoderminae infestation in animals. Thus the present communication records the first attempt of recombinant HyC of *P. silenus* origin for serodiagnosis of GWFI.

## Materials and methods

### Parasite, cells and serum samples

First stage larvae were collected from the subcutaneous tissues of infected goats at the municipal abattoir of Jammu (India), washed with PBS, and identified as per keys of Zumpt^1^ and stored at − 80 °C for RNA isolation. *E. coli* BL21 (DE3)pLysS cells, pET32a(+) vector(Novagen) were used in expression study. Physicoclinical observation was kept as basis for determining reference serum. For sera, blood samples were collected from field and slaughterhouse and marked as positive and negative based on larval detection by palpation in live animals and by carcass examination.Serum samples from animals testing positive for coenurosis, paratuberculosis, enterotoxaemia, oestrosis, coccidiosis, cryptosporidiosis, haemonchosis, monieziosis, fasciolosis, amphistomosis and brucellosis were used in this study.

### *Construction of expression cassette in pET32a(*+*) vector*

Total RNA was isolated from L1 stage larvae of *P. silenus* using RNeasy mini kit (Qiagen, Germany) as per manufacturer protocol. The cDNA was synthesized from total RNA using oligodT primer following the protocol described in cDNA synthesis kit (Revert Aid First strand cDNA synthesis kit, Thermo Scientific). The HyC gene of *P. silenus* was amplified using HyC gene of cattle warble fly (*H. lineatum*) specific expression primers based on the published sequences of HyC (EMBL Accession Number X74306) (AAGGATCCATAATCAATGGATACGAAG and ATCTCGAGTTAAAATATTATACCAGTATTTTG). The gene was amplified using a 25 µL reaction mixture containing 2 µL of cDNA, 2.5 µL of 10X Ex Taq buffer (TaKaRa,USA), 1 µL each of forward and reverse primers (10 pmol/µL), 1 µL of 10 mM dNTP mix, 0.5 µL of Ex Taq DNA Polymerase (TaKaRa, USA), and 17 µL nuclease free water. PCR reaction was carried out at initial denaturation of 94 °C for 5 min followed by 35 cycles of denaturation at 94 °C for 1 min, annealing at 56 °C for 1 min, extension at 72 °C for 1 min and a final extension at 72 °C for 10 min.

The amplified PCR product was analysed by 1% agarose gel electrophoresis. The amplified product was purified using Wizard™ SV Gel and PCR Clean-Up System (Promega, USA), directionally cloned into pET32a(+) expression vector at *BamH*I and *Xho*I restriction enzyme sites and transformed into TOP10 *E. coli* cells. The recombinant clone was selected on LB agar plate containing Ampicillin (100 μg/mL). The recombinant plasmid containing gene of interest was confirmed by colony PCR using gene-specific primers and restriction enzyme digestion. The recombinant plasmid was isolated using Plasmid extraction kit and sent for nucleotide sequencing to Agrigenome Pvt. Ltd (Kerala). The sequence received was primarily analysed using BioEdit software and submitted to Gen Bank. Further, the protein encoding nucleotide sequence was translated in silico using the Edit Sequence program of DNA Star (Laser gene Suite 6.0) and BLASTp (protein–protein BLAST) was performed. The sequence generated here was compared to the reference bovine sequences available in the public domain and aligned using CLC Genomics Workbench (Qiagen)^39^.

The deduced protein sequence of HyC originated from *P. silenus* was aligned with HyC of other Hypoderminae spp. viz *H. lineatum, H. bovis and H. diana* with accession numbers X74306, MK473847, EU999953, respectively. The protein sequences were aligned with CLC genomics platform version 20.01 (Qiagen).

### Expression and purification of recombinant protein

The recombinant HyC plasmid was transformed into *E. coli* BL21(DE3)pLysS host cells and further confirmed by colony PCR. The positive clone was grown in LB broth containing ampicillin and chloramphenicol at 37 °C for overnight. The fresh LB broth was inoculated at 1:100 with overnight culture and incubated at 37 °C at 200 rpm till the culture reached an OD_600_ 0.4–0.6. Henceforth, the expression was induced with 1 mM IPTG and harvested 1 mL culture at hourly interval up to 16 h. The collected fraction was pelleted by centrifugation and level of expression was analysed by SDS-PAGE. The rHyC protein was produced in bulk and the pellet was resuspended into denaturing buffer (8 M Urea) and sonicated at 25% amplitude with pulse of 15 s for 5 min. The lysate was clarified and the supernatant was purified by using Ni–NTA affinity chromatography under denaturing condition (8 M Urea). The Ni–NTA superflow gravity column was equilibriated with denaturing binding buffer containing 8 M urea in phosphate buffer (pH 7.8) followed by binding of clarified lysate with Ni–NTA Superflow® resin (Qiagen). The protein-bound resin was washed in subsequent steps with wash buffer at pH 6.0 and pH 5.3 upon which the bound target protein was eluted with elution buffer (100 mM PB/150 mM NaCl/8 M Urea) at pH 4.0. The eluates were analysed by SDS-PAGE, pooled and dialyzed under decreasing concentrations of urea 6 M, 4 M, 2 M and finally Tris buffer, pH 8.0. The purity of the recombinant protein was analysed by 12% SDS-PAGE gel after staining with Coomassie brilliant blue R-250 stain. The concentration of the recombinant protein was quantified by Bradford protein assay kit (VWR, USA) and stored at − 80 °C.

### Western blotting

Purified recombinant protein was resolved under 12% SDS PAGE and blotted on nitrocellulose membrane (0.45 μm, Biorad) by Mini Trans-Blot^®^ electrophoretic transfer cell (Bio-Rad). The membrane was blocked overnight with skimmed milk powder 5% (SMP) at 4 °C. Thereupon, the membrane was washed thrice with PBS-Tween-20 (0.05%). The individual lanes were cut and incubated with goat warble fly infected serum (1:50), negative control sera and *Oestrus ovis* positive sera (1:50), respectively at 37 °C for 1 h under gentle shaking. Upon triple wash with PBS-T, the membranes were incubated with anti-goat IgG HRP (1:5000 in PBS) for 1 h at 37 °C. The membranes were washed three times and developed with DAB substrate (VWR, USA).

### Optimization of rHyC based iELISA

An indirect-ELISA using rHyC has been optimised and evaluated with known status of positive and negative GWFI sera to detect the anti-HyC antibodies. The optimum concentration of coating antigen, dilution of serum and conjugate, standard checkerboard titration was performed. A total of three different concentration of antigen i.e. 0.5 μg/mL, 0.25 μg/mL and 0.125 μg/mL (100 μL/well), three different dilutions of sera at 1:400, 1:800 and 1:1600 and two dilutions (1:5000 and 1:10,000) of anti-goat IgG HRP conjugate were used in checkerboard titration analysis using 5% SMP as blocking buffer. The maximum differentiation between positive and negative sera (P/N) measured at A_490_was selected for further analysis.

In brief, 96-well microtiter plate (Costar 3590, Corning-USA) were coated with 100 μL of rHyC (0.5 μg/mL) in 0.05 M carbonate bicarbonate buffer (pH 9.6). The plate was incubated at 4 °C overnight. The wells were washed twice with PBS-Tween 20 (0.075%), and then once with PBS, blocked with 5% skimmed milk prepared in PBS (pH 7.4) and incubated at 37 °C for 1 h. After washing, 100 μL of known positive and negative serum sample diluted in PBS (1:400) were added and incubated at 37 °C for 1 h. After washing the plate three times, 100 μL of anti-goat IgG HRP-conjugate (Sigma Aldrich, USA) at 1:10,000 dilution in PBS (pH 7.4) was added to each well and incubated at 37 °C for 1 h. Following washing, 100 μL of O-phenylenediamine dihydrochloride (OPD) substrate (Sigma Aldrich, USA) dissolved in citrate–phosphate buffer (pH 5.0) with 30% H_2_O_2_ was added to individual wells. The reaction was allowed to develop for 10 min in dark and reaction was stopped by adding 50 μL of 1 M H_2_SO_4_ to each well. Absorbance at 490 nm was measured by using microplate reader (iMarkmicroplate reader, Biorad).

### Evaluation of rHyC based iELISA

After optimisation of the iELISA, serum samples with known status (15 positive and 25 negative) were screened in duplicate for determining the cut-off value. The physicoclinical evaluation was taken as a reference to distinguish the positive and negative samples. Negative reference serum samples were received from animals that had never grazed and belongs to Punjab state of North India, where GWFI has never been reported. The cut-off value was evaluated by the Receiver Operating Characteristic (ROC) curve, using MedCalc software, Mariakerke, Belgium^40^. Based on the checkerboard titration results, ROC curves and areas under the curve were plotted, and the sensitivity and specificity of iELISA were calculated using interactive dot diagrams. Cut-off value was calculated according to the Youden index (Youden = Se + Sp − 1) and used for immunodiagnosis of goat warble fly. In order to rule out the cross reactivity of optimised rHyC based iELISA with other economically important parasitic and bacterial diseases of goats, known sera positive for oestrosis, monieziosis, coccidiosis, fasciolosis, haemonchosis, amphistomiosis, coenurosis, cryptosporidiosis, Johne’s disease, brucellosis and enterotoxaemia were also screened.

### Screening of random samples

A total of 421 serum samples collected from municipal slaughterhouses in Jammu and different parts of Union Territory of Jammu and Kashmir namely Jammu, Rajouri, Udhampur, Poonch, Samba,Kathua, Reasi, Kishtawar and Doda were tested by optimized rHyC based iELISA. All the samples were tested in duplicates. The sample showing a difference of more than 0.100 in raw OD values between duplicates were not considered in the present data.

### Ethics approval

All the serum samples used in the study were collected by the research team under informed consent of goat farmers under the guidelines of Institutional Animal Ethics Committee [AU/FVSJ/AGB/17-18/415 Dated: 31-03-2018].

## Results

### Cloning and bioinformatics analysis of rHyc from *P. silenus*

**Figure 1:**
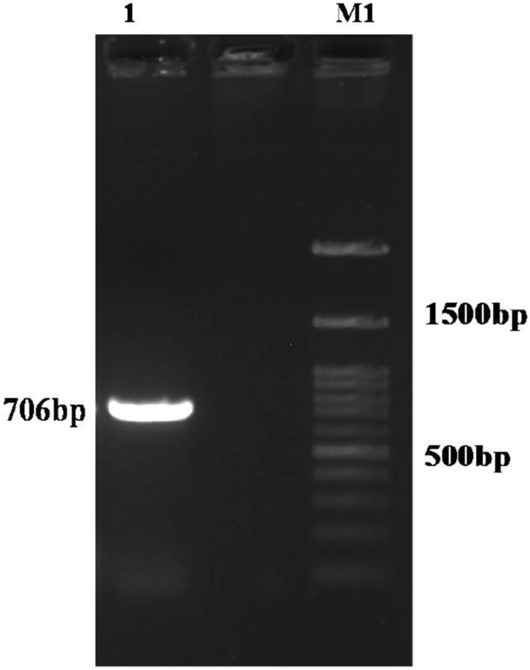
PCR amplification of CDS of hypodermin C gene of *P. silenus*. M1: 100 bp plus DNA ladder, 1: 706 bp PCR amplified product of hypodermin C CDS.

PCR amplification of the HyC from *P. silenus* was obtained by using cDNA template produced from L1 larval instars with oligodT primers. The amplified product of expected size 706 bp was confirmed on 1% agarose gel electrophoresis (Fig. 1). The gel purified PCR product was cloned into pET32a( +) vector at *BamH*I and *Xho*I restriction enzyme sites and transformed into TOP10 *E. coli* cells. The transformants were screened on LB agar plates containing ampicillin. The recombinant plasmid was characterised by colony PCR and RE digestion that confirmed the presence of target gene and its orientation into the vector.

**Figure 2:**
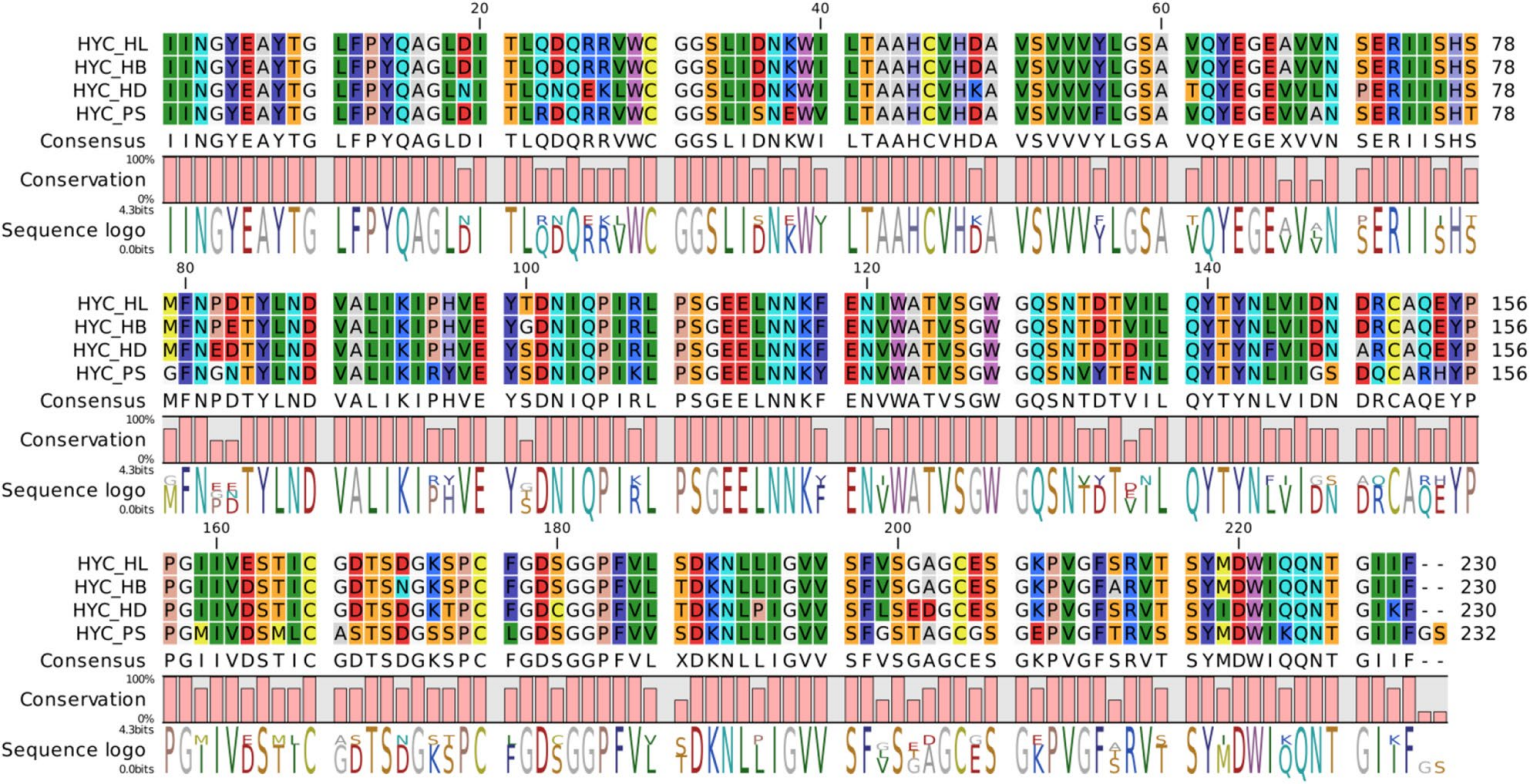
Protein sequence alignment of HyC derived from *P. silenus* (HYC_PS) with HyC from *H. lineatum* (HYC_HL), *H. bovis* (HYC_HB) and *H. diana* (HYC_HD).

The recombinant plasmid was isolated, sequenced and the gene sequence was submitted to GenBank (Accession number MZ407908). Sequence similarity searches in Blastn revealed that the HyC from *P. silenus* (HyC_PS) was 86% identical to HyC of *H. lineatum* and *H. bovis*. The alignment of HyC_PS with HyC protein reported from *H. lineatum* (HyC_HL), *H. bovis* (HyC_HB) and *H. diana* (HyC_HD) revealed 18.695% variation in the polypeptides, with 43 substitutions out of total 230 amino acids (X74306.1) (Fig. 2). The HyC_PS with 232 residues with a predicted molecular weight of 25.150 kDa contains 22 strongly acidic, 12 strong basic, 79 hydrophobic residues and 78 polar amino acid residues with a predicted pI of 4.569. The molecule contains all the six cysteine residues as shown in sequence logo, at conserved position resulting in three disulphide bonds. The conserved residues of catalytic triad as His-45, Asp-88, Ser-180 are also present in the protein representing the functional feature of serine proteases (Fig. 2).

Secondary structure prediction analysis indicated the trypsin domain structure for HyC with presence of three chymotrypsin peptidase regions in the HyC amino acid sequence. Secondary structure analysis also revealed that HyC_PS consists of 9% alpha-helix, 43% beta-strand and 16% unstructured coil regions, respectively^41^.

### Expression of rHyC and its characterisation

The verified recombinant clone with HyC gene in BL21 cells was used for expression study. The expression of protein was induced through 1 mM IPTG at 37 °C and the samples collected at hourly interval were analysed by SDS-PAGE to check the maximum level of expression for the construct. The different fractions were compared for expression with the uninduced sample showed about 45 kDa of protein overexpressed upon 6 h post induction, maximally. The protein separated in SDS-PAGE was transferred on nitrocellulose membrane (NCM) for blotting and expressed recombinant HyC protein was confirmed by Ni–NTA HRP conjugate.

### Purification and immunoreactivity of rHyC

**Figure 3:**
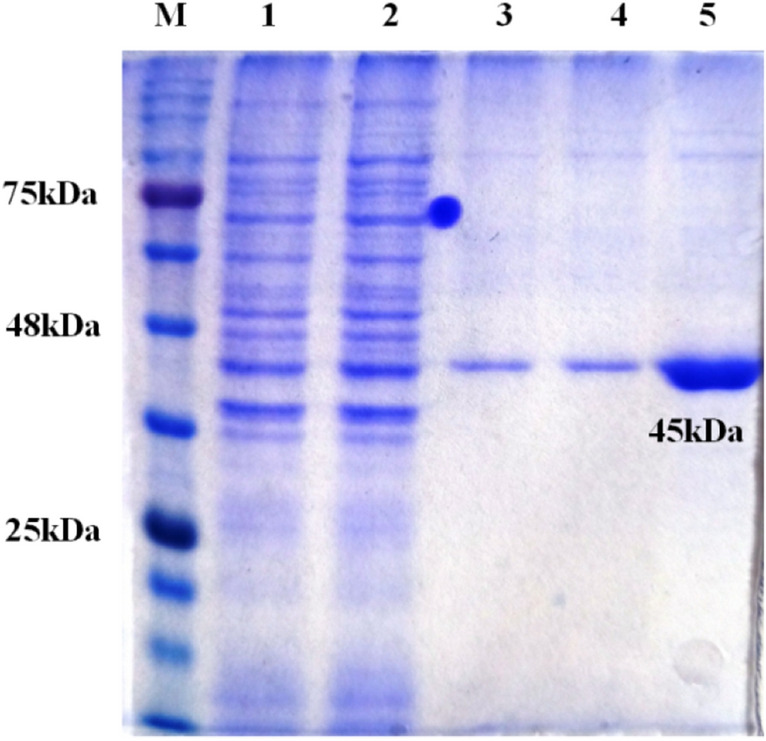
Ni–NTA purification of recombinant hypodermin C fusion protein (45 kDa) M: Marker, Lane 1, 2: elutes of wash buffer Lane 3, 4, 5: elutes of purified protein.

**Figure 4:**
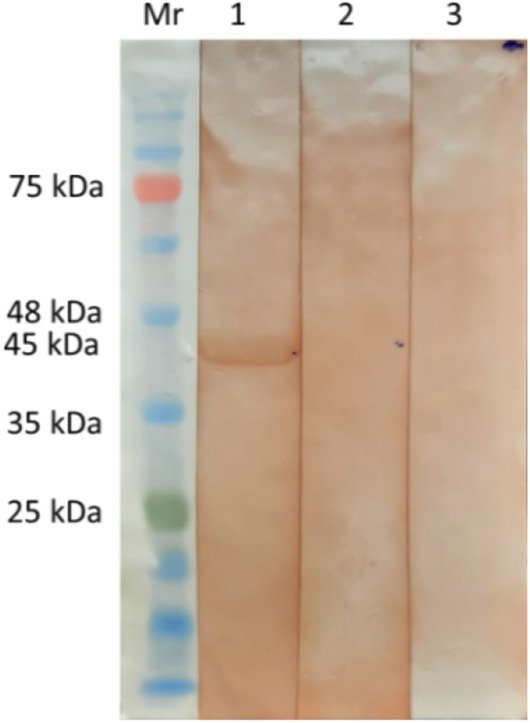
Western blotting of rHyC protein M: Marker 1: immunoreactivity with natural warble fly positive goat serum, 2: commercial sterile goat serum, 3: *Oestrus ovis* positive serum.

After optimisation, expression was scaled up by increasing the culture volume and purified under denaturing condition using Ni–NTA affinity chromatography. The eluted fractions were analysed by SDS-PAGE that indicated the presence of single band with about 45 kDa size with near homogeneity (Fig. 3). The yield of expressed fusion protein was at 2.8 mg/L of induced culture. The antigenicity of purified recombinant protein was confirmed by immunoblotting using GWFI positive, negative serum and Oestrosis positive sera. Recombinant HyC specificity was confirmed to GWFI serum. A distinct band of 45 kDa was observed with the expressed protein reacting strongly and specifically with the GWFI positive serum. No reactivity was observed with goat negative and oestrosis positive sera (Fig. 4).

### Optimisation and evaluation of rHyC iELISA

**Figure 5:**
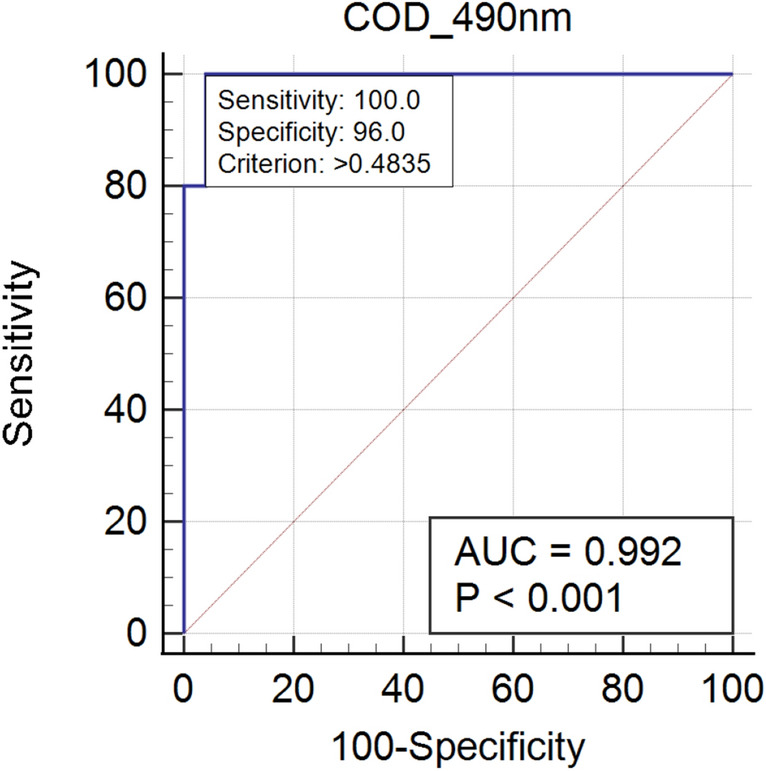
ROC curve analysis representing sensitivity and specificity of recombinant hypodermin C (rHyC) indirect ELISA.

**Figure 6:**
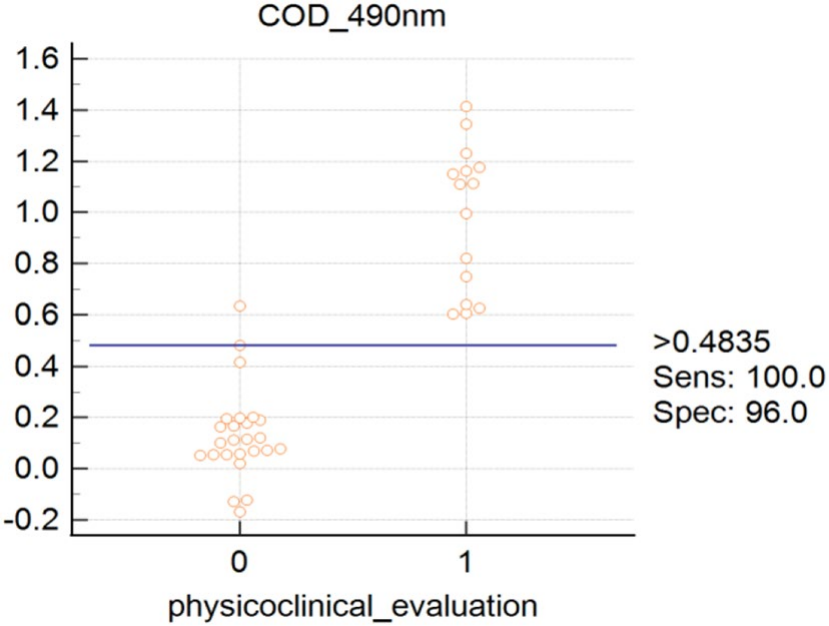
Dot plot for different serum samples and cut-off for the recombinant hypodermin C (rHyC) indirect ELISA by ROC analysis.

Checkerboard titration revealed that at antigen concentration of 0.5 μg/mL (100 μL/well), serum dilution of 1:400, conjugate dilution of 1:10,000 with 5% SMP as blocking buffer were found to give maximum difference in the reactivity between positive and negative serum. ROC analyses using sera from 15 infested and 25 non- infested goats revealed an area under curve (AUC) of 0.992 (*P* < 0.001) for rHyC iELISA. Relative diagnostic sensitivity and specificity calculated by interactive dot analysis of MedCalc software were 100% and 96%, respectively (Fig. 5). A cut off value (OD) of 0.4835 for rHyC iELISA was fixed from the ROC curve and interactive dot diagram (Fig. 6) and used for serological screening of the random samples.

**Figure 7:**
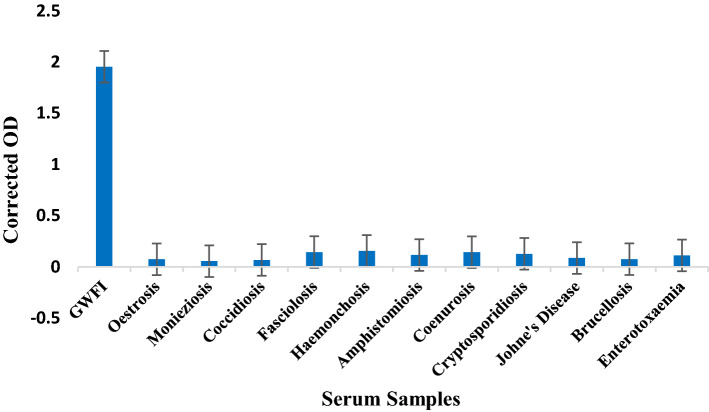
Cross reactivity of optimized ELISA with most common parasitic and bacterial diseases of goat.

**Figure 8:**
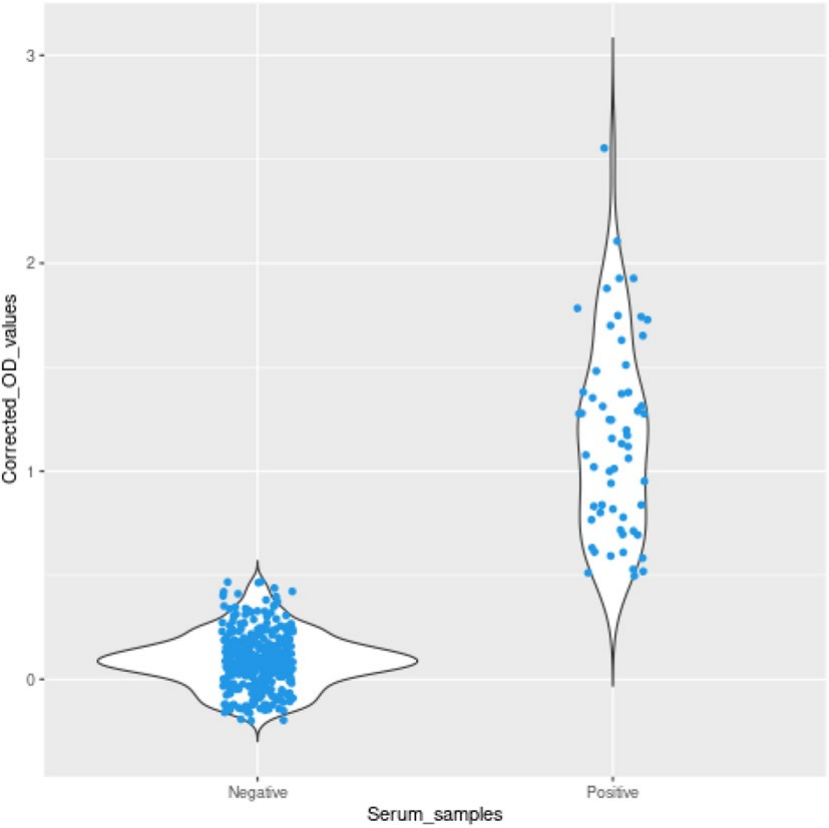
Distribution of ELISA results by sample classification as negative and positive.

The expressed protein showed strong reactivity with GWFI positive serum whereas it did not show any significant reactivity with other economically important parasitic and bacterial diseases of goat (Fig. 7). Further, screening of random serum samples collected from different parts of Union Territory of Jammu and Kashmir, North India were tested with optimised ELISA and the corrected OD values (Mean OD of duplicates − Mean OD of antigen blank) were calculated to differentiate between infested and healthy animals. Out of 421 serum samples screened through optimised iELISA for detecting anti-HyC antibodies, 59 were found to be positive and 362 tested negative for GWFI (Fig. 8).

## Discussion

Goat warble fly disease is the cause of major economic losses to goat farmers with high prevalence in Mediterranean and Indian subcontinent. GWFI has been reported with varying prevalence rates in different countries. In North India, the disease has been reported to have a prevalence rate varying between 13 and 56.5% in Jammu region whereas the prevalence varies in Pakistan (5–75%), Turkey (53–94%), southern Italy (20–90%), Iran (7–19%) and Jordan^4,6,7,11,12,43^. The diagnosis of GWFI is based on physicoclinical examination of the suspected animal for presence of subcutaneous warble, caused by late stage of L2 and L3 larval instars of *P. silenus*, on dorsal palpation or carcass examination upon slaughter. In addition to physical observation, serodiagnosis has been used to detect antibodies against larval antigens. Physicoclinical detection of GWFI upon slaughter or field observation is a setback in early detection of infestation at L1 stage of larvae in the first 3 months of infestation by parasite. Goat warble fly, *P. silenus* causes subcutaneous myiasis with similar characteristics as exhibited by *H. lineatum* and *H. bovis* in cattle and other ruminants. *P. silenus* in family hypodermatinae also exhibit immunoreactivity with HyC antigen from *H. lineatum* and *H. bovis*^19^. Serological detection of infestation is facilitated by an immunoassay detecting the anti-*P. silenus* antibody in host (goat) at the first month of infestation^37^, similar to cattle antibody kinetics corresponding to *H. lineatum* and *H. bovis* for cattle hypodermosis^30,37,43^.

Hypodermin A, B and C are serine proteases from Hypoderminae insects which are the major immunogens in case of larval infestations. Hypodermins are reported to show overexpression in L1 larval instar as compared to L2 and L3 stages^32^. Hypodermin C has been established as the major antigen for serological detection of hypoderminae myiasis and shows cross-reactivity among Hypoderminae members (*H. bovis*, *H. lineatum*, *H. tarandi*, *H.actaeon, H. sinense* and *P. silenus*) due to shared epitopes as evident by several studies of cross reactivity and ELISA^19,21,35,38,44,45^. The HyC derived from *P. silenus* has shown several substitutions interspersed in the whole sequence. On comparison with other serine proteases reported from *H. lineatum*, *H. bovis* and *H. diana*, the HyC presents all the conserved features attributed to serine protease activity such as conserved cysteine residues and the catalytic triad at the designated sites (His-45, Asp-88, Ser-180)^15,28,32^. Although, the HyC protein derived from *P. silenus* show considerable variations in residues (18.695%, 43 substitutions/230 aa), the three dimensional structure shows strong homology in the predicted structure as compared to that of HyC from *H. lineatum*^16^.

ELISA has been promoted as surveillance tool in diagnosis of warble fly in various countries^19,21,46–49^. It is preferred over palpation or examination of carcass at slaughter^49^. Serology provides more sensitive detection of exposure to warble fly myiasis as a number of animals might resolve larvae over a period and can only present serological detection^49^.Thus, ELISA provides a tool of warble fly infestation study in occult stages as well as in case of early resolution of the larvae before warble development on the dorsum in case of GWFI. The antibody kinetics against *H. lineatum, H. bovis, P. silenus, and H. tarandi* in their hosts have revealed that it takes about one month to reach antibody peaks in the host sera, which implicates the utility of ELISA as diagnostic tool at L1 stage of infestation^20,29,30,37^.

Several in-house ELISA assays are used in different countries which are primarily based on native antigen derived from *H. lineatum and H. bovis* larvae. Native antigen and purified antigen preparations provide challenge in standardisation due to purification and requirement of larvae repeatedly. Recombinant protein may serve as antigen for better reproducibility and standardisation for a diagnostic test. Also, since the parasite availability for native antigen is decreasing and fluctuating upon progressive control measures in many countries, recombinant proteins serve as constant source of antigen for diagnostic evaluation of the population^33^. Recent study has provided rHyC based indirect ELISA derived from *H. bovis* for the diagnosis of hypodermosis in cattle^28^ whereas another study developed in-house ELISA protocol for the detection of GWFI^9^ which are based on the use of a larval crude lysate as antigen. A summary of selected ELISA for Hypoderminae myiasis detection is provided in Table 1. In natural infestations, larval development is not synchronous as individual hosts may have multiple oviposition events and this apparently causes episodic release of HyC limiting the utility of antigen-capture ELISA^31,50,51^. On the other hand, in case of competitive ELISA, sample dilution is permitting lower coverage of the herd surveillance in case of pooled sampling^36^.

**Table 1:** Summary of selected in-house immunoassays developed for the diagnosis of Hypoderminae species.

ELISA	Antigen used	Target analyte	Warble species	Host	Sample	Dilution factor	Reference
Indirect ELISA	Crude lysate (*H. bovis*)	Anti-*Hypoderma* Ab	*H. bovis /H. lineatum*	Cattle	Sera	–	^23^
Indirect ELISA	Secretory and somatic (*H. lineatum*)	Anti-*Hypoderma* Ab	*H. lineatum*	Cattle	Sera	1:200	^25^
Competitive ELISA	Native antigen (*H. lineatum*)	Anti-*Hypoderma* Ab	*H. lineatum*	Cattle	Sera	1:20–1:40	^36^
Antigen capture ELISA	–	Circulatory HyC	*H. lineatum*	Cattle	Sera	1:10	^30,31^
Indirect ELISA	Recombinant HyC *(H. lineatum)*	Anti-HyC Ab	*H. lineatum*	Cattle	Sera	1:100	^26^
Indirect ELISA	Recombinant HyC (*H. bovis*)	Anti-HyC Ab	*H. bovis*	Cattle	Sera	1:200	^28^
Competitive ELISA	Crude larval Ag (native Ag)	Anti-*Przhevalskiana* Ab	*Przhevalskiana* spp.	Goat	Sera	1:200	^9^
Indirect ELISA	Recombinant HyC (*P. silenus*)	Ant-HyC Ab	*P. silenus*	Goat	Sera	1:400	Present study

The present study provides first molecular characterisation of HyC protein, the major antigen for serodiagnosis of warble fly myiasis, in case of goats, caused by *P. silenus*. The study characterised the HyC of *P. silenus* by cloning and heterologous expression in *E. coli* and assessed its immunoreactivity with GWFI positive sera along with other economically important goat diseases.

The HyC derived from *P. silenus* was amplified with gene specific primers and cloned into pET 32a(+) vector. The expected size of target protein is about 25 kDa whereas the complete construct used in this study presents around 45 kDa product as a fusion protein with thioredoxin tag and the same size was obtained as expected in SDS-PAGE analysis upon induction. The optimum level of expression was achieved at 6 h post-induction after which there was no improvement in the expression level. Purification was done under denaturing conditions with 8 M Urea in PBS with elution based on pH gradient in acidic condition. At a yield of 2.8 mg/L of induced culture, the recombinant fusion protein may be sufficient to screen approx. 2,800 serum samples in duplicate with the optimised protocol. The purified HyC protein reacted strongly with GWFI positive serum in ELISA format at antigen concentration of 0.5 μg/mL (100 μL/well) at 1:400 dilution of serum identified by checkerboard titration. The present ELISA showed no cross reaction with any other major parasitic and bacterial diseases of goats. The rHyC of *P. silenus* as an antigen in the ELISA provided a diagnostic sensitivity and specificity of 100% and 96% respectively, for serodiagnosis of goat warble fly disease The other iELISA established with rHyC derived from *H. lineatum* exhibited sensitivity of 85% and specificity of 98.2% whereas iELISA based on rHyC from *H. bovis* has shown sensitivity of 90% and specificity of 100%^25,27^. Also, the study comparing utility of native HyC (nHyC) and rHyC as antigen in ELISA for detection of anti-Hypoderma antibodies has shown a sensitivity of 95.8% and specificity of 95.7% for rHyC and 98.2% sensitivity as well as specificity for nHyC based iELISA^32^. On the other hand, competitive ELISA for detection of hypodermosis showed a sensitivity and specificity of 92.5% and 98.5%, respectively^36^.

The iELISA based on rHyC is optimised with a high sample dilution rate at 1:400 for GWFI serum samples. This provides an opportunity for a standardised test as a tool for mass surveillance of GWFI at national eradication program. Also, the high standard dilution rate permits scope of using pooled serum samples for the assessment of herd screening for GWFI^52,53^. It has also been used for the screening of random serum samples from different parts of Union Territory of Jammu and Kashmir, North India. The optimised assay is highly specific and sensitive for detection of antibodies against goat warble fly disease.

ELISA test has been adopted for mass epidemiology of hypodermosis as a tool of monitoring at national level eradication programs in countries like France, the UK, Belgium and Germany^37^. ELISA has proven an effective tool to establish antibody kinetics in both bovine hypodermosis and GWFI. This has further assisted the monitoring initiatives to facilitate appropriate timing of sample collection according to particular geographical and agroclimatic zones^8,9,19,54^. Durable control of GWFI requires repetitive monitoring for the detection of any reappearance of the disease in any geographical region. Serological monitoring is vital tool for aid in policy implementation of control and eradication programmes through the use of systemic insecticides^52,53^. As it is evident by immunological studies that Hypoderminae species do not provide complete protective immunity, it is plausible that constant monitoring and surveillance along with prophylactic treatment remains the practical solution for durable control of goat warble fly as has been observed in cattle hypodermosis^49,51^.

## Supplementary Information

Supplementary Information 1.

Supplementary Information 2.

Supplementary Information 3.

Supplementary Information 4.

## Data Availability

All data generated or analysed during this study are included in this published article (and its supplementary information files). The sequences used for comparison are available under the accession numbers: X74306; MK473847; EU999953; MZ407908 from NCBI database at https://www.ncbi.nlm.nih.gov/.
