# Supplementary material for: First report of Przhevalskiana silenus derived recombinant hypodermin C based indirect ELISA for serodiagnosis of goat warble fly myiasis
Source: Sci Rep. 2022 Aug 4;12:13440. doi: 10.1038/s41598-022-17760-5 (PMC9352896; doi:10.1038/s41598-022-17760-5)
Supplement: Supplementary file 4 — Supplementary Information 4. [file 41598_2022_17760_MOESM4_ESM.docx]

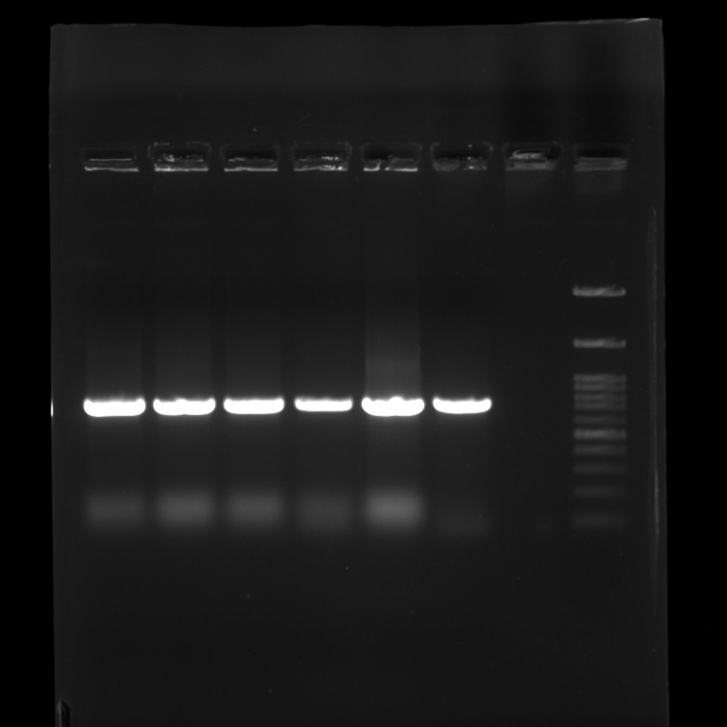


***Figure 1: Whole gel picture of*** ***PCR amplification of CDS of Hypodermin C gene of P. silenus. M1: 100bp plus DNA ladder, 1: 706bp PCR amplified product of hypodermin C CDS***

.
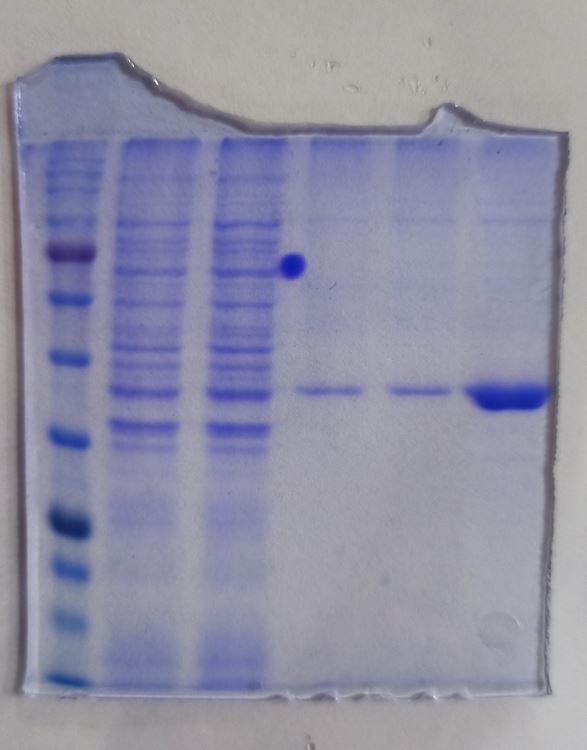


***Figure 3: Whole gel picture of Ni-NTA purification of recombinant Hypodermin C fusion protein (45kDa) M: Marker, Lane 1, 2: elutes of wash buffer Lane 3,4,5 : elutes of purified protein. The image region used in manuscript is highlighted with white box.***


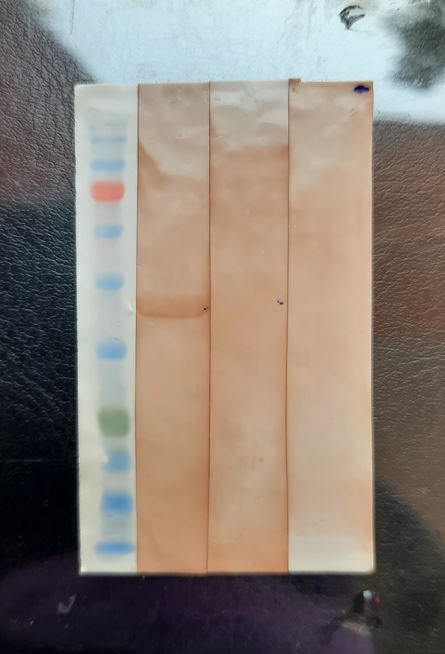


***Figure 4: Original picture of western blotting of rHyC protein M: Marker 1: Immunoreactivity with natural warble fly positive goat serum, 2: Commercial sterile goat serum, 3: Oestrus ovis positive serum [The individual lanes were cut to treat with GWFI positive serum, Commercial sterile goat serum, Oestrus ovis positive serum individually and thereupon arranged in order shown above]***
